# Assessment of Medication Adherence to Anti-tuberculosis Treatment Among Patients With Tuberculosis at a Tertiary Care Hospital in South Gujarat, India

**DOI:** 10.7759/cureus.95495

**Published:** 2025-10-27

**Authors:** Savan Ajmera, Nrupal Patel, Mayur Chaudhari, Grinish Tamakuwala, Milan Dangar, Tushar Talavia, Lay Shah, Chetankumar Acharya

**Affiliations:** 1 Department of Pharmacology, Government Medical College, Surat, Surat, IND; 2 Department of Pulmonary Medicine, Government Medical College, Surat, Surat, IND

**Keywords:** 8-item morisky medication adherence scale (mmas-8), adverse drug reactions (adrs), factors affecting medication adherence, tuberculosis, medication adherence

## Abstract

Background

Tuberculosis (TB) remains a major public health challenge, especially in developing countries like India. A successful TB treatment relies heavily on good medication adherence, which helps to prevent complications such as drug resistance and poor treatment outcomes. Multiple factors influence medication adherence, including socioeconomic, patient-related, and therapy-related aspects.

Objectives

This study aims to assess the level of medication adherence and factors affecting medication adherence in TB patients during treatment.

Methodology

A cross-sectional observational study was conducted among 223 TB patients (≥18 years) who had received more than two months of anti-TB treatment in a tertiary care hospital. Medication adherence was assessed using the 8-item Morisky Medication Adherence Scale (MMAS-8). Data were analysed based on demographic and clinical variables using IBM SPSS Statistics for Windows, V. 30.0 (IBM Corp., Armonk, NY, USA).

Results

Among 223 patients, 126 (56.5%) were male and 97 (43.5%) were female with a mean age of 35.08 ± 13.28 years. Using MMAS-8 to analyse medication adherence levels, 150 (67%) patients showed high medication adherence, 41 (18%) medium, and 32 (15%) low. High medication adherence was more prevalent among 70 (72.16%) females as compared to 80 (63.49%) males. Medication adherence was similar between 92 drug-sensitive TB (67.6% high medication adherence) and 58 drug-resistant TB (66.7% high medication adherence) cases. The most common factors affecting medication adherence were difficulty in procurement of medicines, lack of family support, and misunderstanding of treatment schedules. The most frequently reported adverse drug reaction was gastrointestinal upset (60, 46.9%), followed by neuropathy (22, 17.2%) and arthralgia (15, 11.7%).

Conclusion

The study showed good medication adherence among TB patients, supported by programmatic measures. Maintaining and enhancing medication adherence still requires addressing financial obstacles, enhancing medication availability, and monitoring and managing adverse reactions.

## Introduction

Tuberculosis (TB) is a chronic granulomatous disease, caused by *Mycobacterium tuberculosis* bacteria, which is a major health-related problem worldwide but specially in developing countries. Every year, there are more than 10 million new TB cases, of which 0.6 million are caused by drug-resistant *Mycobacterium tuberculosis*. According to the India TB Report 2024, the estimated number of TB cases in India in 2023 was 2.78 million, and the estimated mortality was 0.32 million [1]. A total of 1.3 million people died from TB in 2022 (including 167,000 people with HIV). Worldwide, TB is the second leading infectious killer after COVID-19 (above HIV and AIDS). TB is an infectious disease that most often affects the lungs but also affects the bones, brain, lymph nodes, abdominal organs, and spine. It spreads through the air when infected people cough, sneeze, or spit. It is a curable disease if treatment is received quickly and appropriately. So, rapid and accurate diagnosis and the use of effective anti-TB treatments are priority tools to minimize morbidity, mortality, and the spread of TB among the population [2]. 

Medication adherence is defined by the World Health Organization (WHO) as "the extent to which a person's behaviour taking medication, following a diet, and/or executing lifestyle changes corresponds with agreed recommendations from a healthcare provider" [3]. It includes the initiation of the treatment, implementation of the prescribed regimen, and discontinuation of the pharmacotherapy.

Good medication adherence reduces the complications due to TB like the emergence of multidrug-resistant TB (MDR-TB), extensively drug-resistant TB (XDR-TB), prolonged infectiousness, poor TB treatment outcome [2], and reduction in mortality rate due to TB. However, medication adherence to TB treatment is a serious problem because the results are still far from what is expected.

Poor medication adherence has multifactorial causes that need to be understood before interventions can be designed to improve medication adherence. According to WHO, there are multiple factors leading to poor medication adherence, normally classified into five categories: socioeconomic factors, therapy-related factors, patient-related factors, condition-related factors, and health system/healthcare team (HCT)-related factors [4].

The patient's incapacity or refusal to take TB drugs as directed by a healthcare provider is known as medication non-adherence to therapy. A suitable intervention can be customized to enhance each patient's medication-taking behaviour based on whether the medication non-adherence is primary (starting pharmacotherapy) or secondary (following the recommended regimen) and the circumstances contributing to it. Primary medication non-adherence is when a patient doesn't start a new medication by not purchasing the prescription given by the doctor. Thus, it is about not starting the medication in the first place, while the secondary medication non-adherence occurs when the patient purchases the prescription but doesn't take the medication correctly or regularly or interrupts therapy for more than two months. Irregular medication not only affects the clinical outcome but also affects the financial outcome of the health system [4].

Mere case notification, availability of anti-TB medications, and passive patient observation are not sufficient to ensure adherence and prevent missed follow-up in TB management. Effective therapy requires a comprehensive understanding of the multifactorial determinants influencing a patient's ability to tolerate treatment. Interventions must specifically address these factors to minimize non-adherence and reduce the risk of treatment interruption [5].

Despite the availability of effective anti-TB therapy and implementation of the National TB Elimination Programme (NTEP), poor medication adherence remains a major challenge to achieving successful treatment outcomes and controlling transmission. Non-adherence contributes to treatment failure, relapse, prolonged infectivity, and the emergence of MDR-TB, thereby undermining public health efforts. This study aims to assess the level of medication adherence and factors affecting medication adherence in TB patients during treatment.

## Materials and methods

The present study was designed as an observational study and was conducted in the Department of Respiratory Medicine at Government Medical College, Surat, in Surat, India. The study population comprised both outpatient and inpatient individuals attending the department during the study period. The total duration of the study was 15 months, which included nine months of data collection, three months of data analysis, and three months for writing and compilation. A purposive sampling technique was employed for subject selection. All patients attending the Outpatient Department (OPD) and those admitted to the Respiratory Medicine ward at New Civil Hospital, Surat, who fulfilled the inclusion criteria, were enrolled until the desired sample size was achieved.

Selection criteria 

Inclusion Criteria

Included were patients diagnosed with pulmonary TB (PTB) and extrapulmonary TB (EPTB) and who have received anti-TB drugs for at least two months aged 18 years or more, of either gender, and willing to give written informed consent voluntarily.

Exclusion Criteria

TB patients admitted to the ICU and TB patients with psychiatric illness were excluded from the study.

Sample size

According to a previous study, the prevalence of adherence to anti-TB medication was 64.2% [6]. Based on the OpenEpi software (Dean AG, Sullivan KM, Soe MM. OpenEpi: Open Source Epidemiologic Statistics for Public Health, Version. www.OpenEpi.com, updated 2013/04/06) with a 95% confidence level, the sample size of 223 was calculated for this study.

Study measures

The 8-item Morisky Medication Adherence Scale (MMAS-8) was used to assess the level of medication adherence in TB patients [7], and a pre-validated questionnaire was also used to assess the factors affecting medication adherence (see Appendices).

A face-to-face interview was conducted by the investigators by using a pre-validated questionnaire. The information was collected about the patient's general information, treatment details, and treatment outcome. Adherence was measured using the MMAS-8. It is a self-reporting tool to assess medication-taking behaviour. MMAS-8 consists of a set of eight questions with a yes or no answer. Items 1-7 offer "Yes" or "No" responses, with "0" point for every "Yes" response and a "1" point for every "No". While item 8 included a 5-point Likert response option wherein a patient's score was "0" if they chose response "0" and "1" if they chose response "4". The responses "1, 2, 3" are graded as "0.25, 0.50, 0.75", respectively. The total score ranged from 0 to 8. Furthermore, each patient was classified as high, moderate, and low adherence to treatment if the MMAS-8 score was "8", "6 to <8", and "<6", respectively.

Ethical clearance

The study protocol was reviewed and approved by the Research Review Committee of Government Medical College, Surat (approval number: GMCS/STU/RRC-2/Approval/8723/24; date: 16/04/2024).

Statistical analysis

Data were analysed based on demographic and clinical variables using IBM SPSS Statistics for Windows, V. 30.0 (IBM Corp., Armonk, NY, USA).

Cross-tabulations were prepared for various variables, and an association was tested between related variables.

Summary statistics like mean and standard deviation were calculated. Different graphs and charts were prepared for data presentation at appropriate places.

Univariate Analysis

Univariate analysis was done for quantitative variables using mean and standard deviation. The categorical variable was described using a proportion. Appropriate graphs were prepared.

Bivariate Analysis

Association of important parameters with various outcomes was studied using bivariate analysis. Chi-squared tests of significance were applied for different variables.

## Results

**Figure 1:**
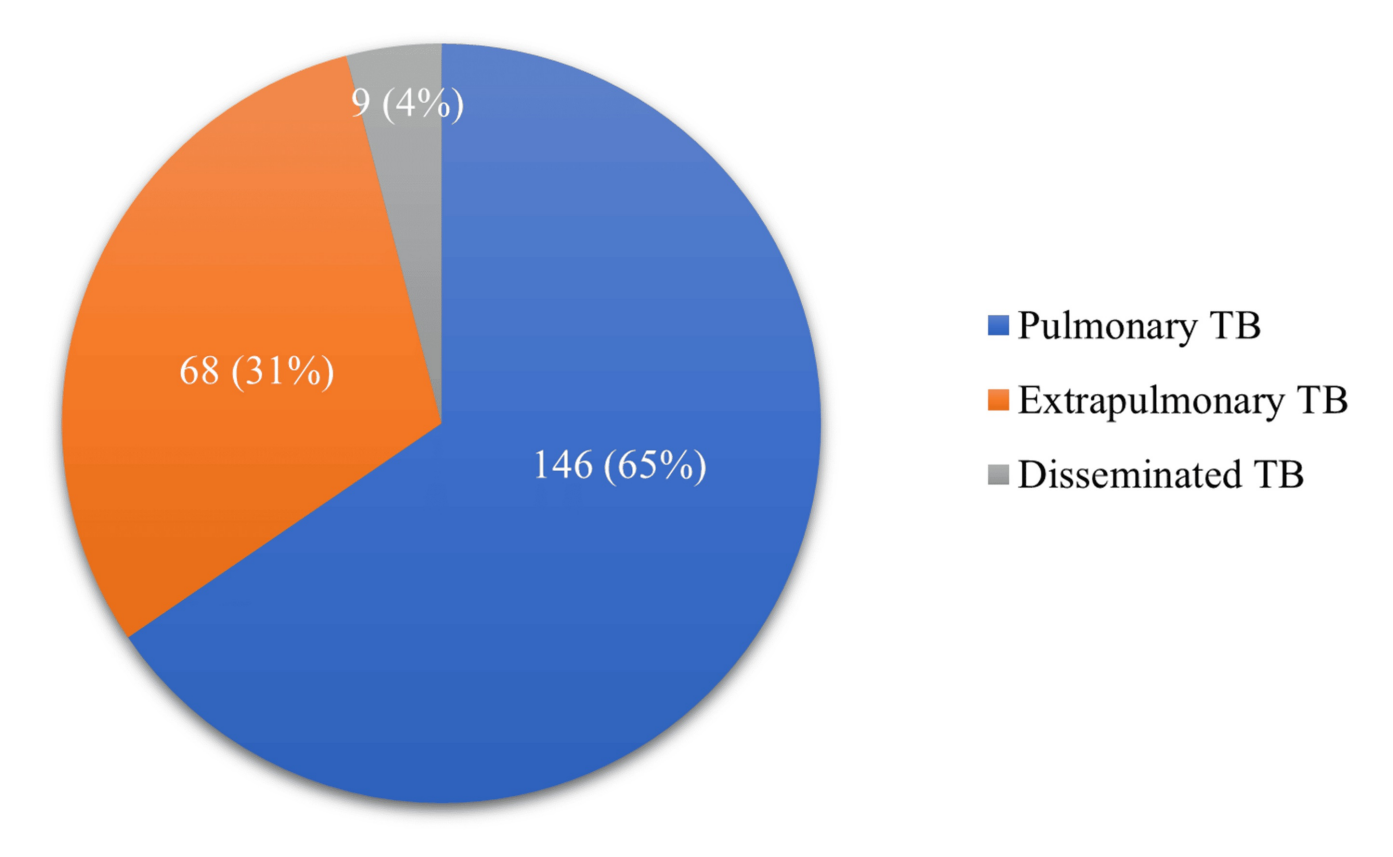
Distribution of TB diagnosis TB: tuberculosis

A total of 223 TB patients were interviewed and assessed, comprising 126 (56.5%) males and 97 (43.5%) females. The mean age of patients was 35.08 ± 13.28 years. Among them, 78 (35%) followed by 74 (33%) patients belonged to the age groups of 28-37 years and 18-27 years, respectively, with a declining trend observed in older age groups. The diagnosis breakdown revealed that 146 (65%) patients had PTB, 68 (31%) had EPTB, and nine (4%) had disseminated TB (Figure 1).

**Figure 2:**
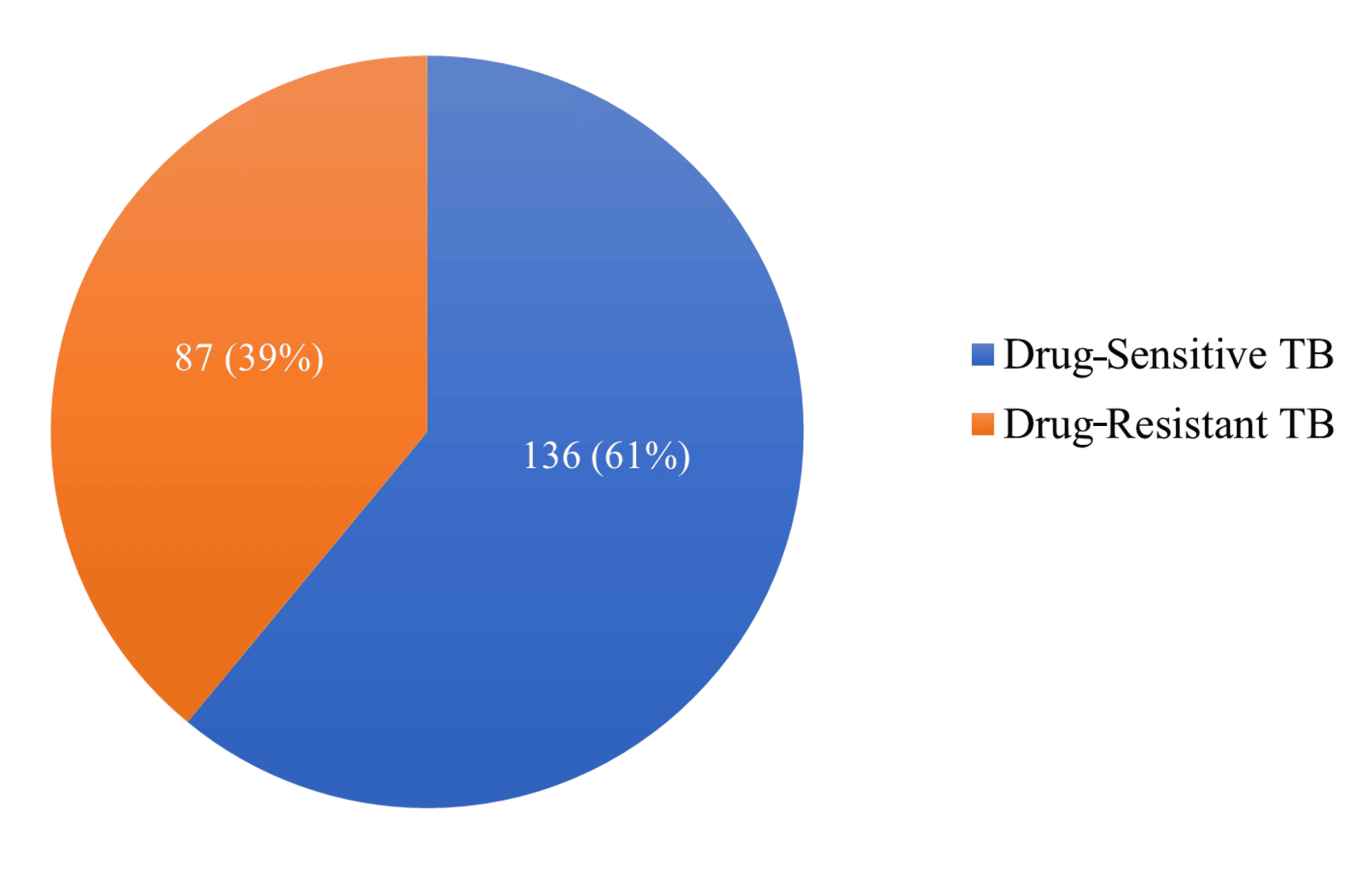
Distribution of TB diagnosis TB: tuberculosis

Out of 223 patients, 136 (61%) were diagnosed with drug-sensitive TB (DS-TB), while 87 (39%) had drug-resistant TB (DR-TB), which included 66 (75.8%) with MDR-TB, 14 (16%) with rifampicin resistance, three (3.4%) with isoniazid resistance, two (2.3%) with isoniazid and rifampicin resistance, and two (2.3%) with XDR-TB (Figure 2).

**Figure 3:**
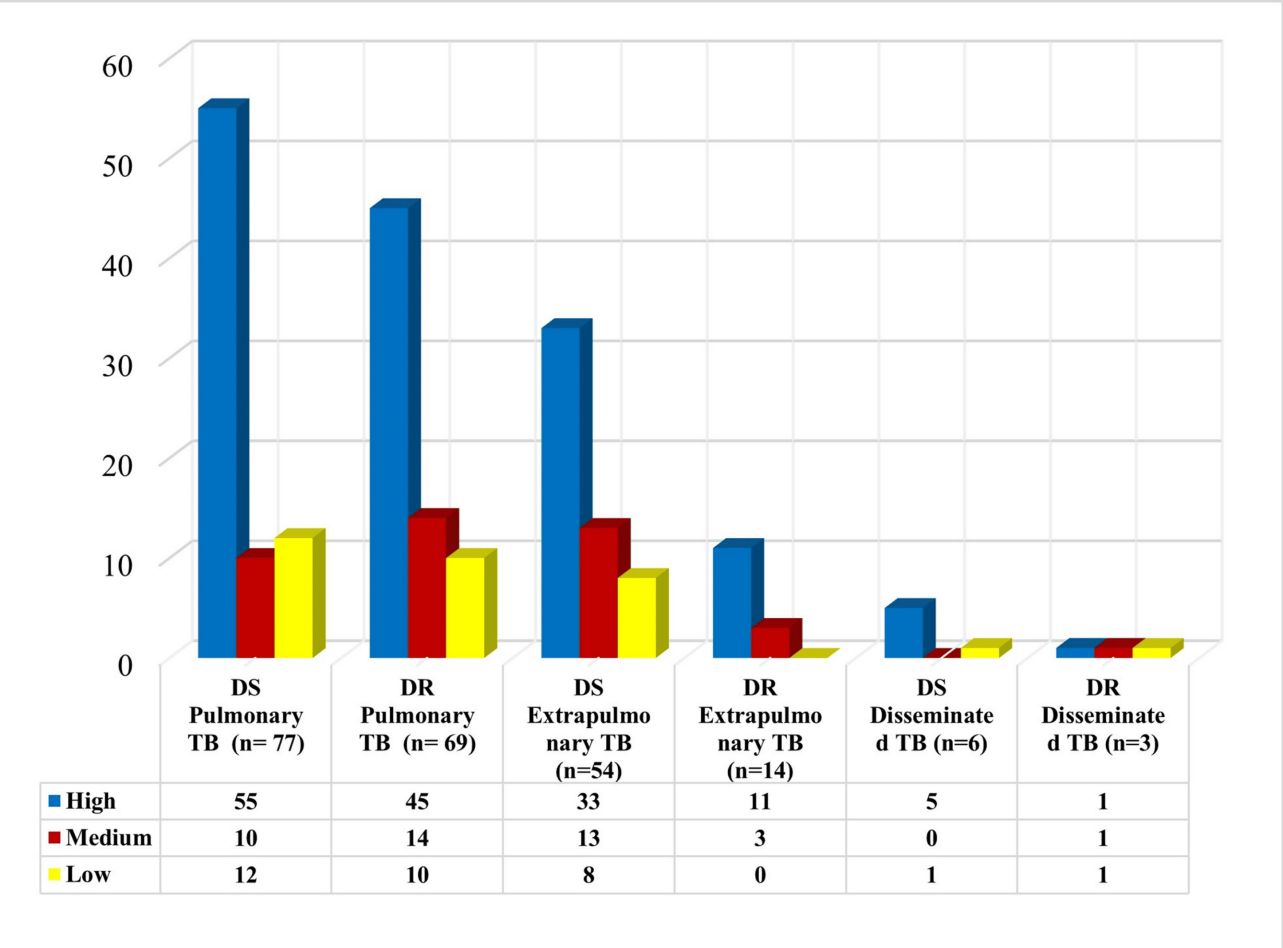
Level of medication adherence among different types of TB TB: tuberculosis; DS: drug-sensitive; DR: drug-resistant

Medication adherence levels were analysed, with MMAS-8, in which 150 (67%) patients showed high, 41 (18%) medium, and 32 (15%) low medication adherence. High medication adherence was more prevalent among 70 (72.16%) females as compared to 80 (63.49%) males. Low medication adherence was observed more in males (27, 21.42%) than in females (five, 5.15%). When stratified by TB type, patients with DS-TB (n=136) had a higher proportion of high medication adherence (92, 67.6%), whereas among those with DR-TB (n=87), 58 (66.7%) exhibited high medication adherence. Medium and low medication adherence were seen in 23 (16.9%) and 21 (15.4%) patients with DS-TB, respectively, and 18 (20.7%) and 11 (12.6%) patients with DR-TB, respectively. A mean MMAS-8 score of 7.25 ± 1.51 for DS-TB and 7.18 ± 1.54 for DR-TB was noted. Among PTB drug-sensitive (PTB-DS) cases (n=77), 55 patients (71.4%) showed high, 10 (13%) medium, and 12 (15.6%) low medication adherence. In PTB drug-resistant (PTB-DR) cases (n=69), 45 patients (65.2%) exhibited high, 14 (20.3%) medium, and 10 (14.5%) low medication adherence. Among EPTB drug-sensitive (EPTB-DS) cases (n=54), 33 patients (61.1%) had high, 13 (24.1%) medium, and 8 (14.8%) low medication adherence. Similarly, in EPTB drug-resistant (EPTB-DR) cases (n=14), 11 (78.6%) and three (21.4%) had high and medium medication adherence, respectively. For disseminated TB drug-sensitive (n=6), five (83.3%) and one (16.7%) showed high and low medication adherence, respectively. Finally, for disseminated TB drug-resistant cases (n=3), one patient each (33.3%) had high, medium, and low medication adherence, respectively. Overall, high medication adherence was most frequently observed among all TB types, with the highest percentage among disseminated TB drug-sensitive cases and the lowest among EPTB-DS patients, indicating a generally good trend in medication adherence among the study population (Table 1, Figure 3).

**Table 1:** Results of the analysis of the relationship of some variables to level of medication adherence in TB patients in taking anti-TB drugs *A p-value of <0.05 is considered statistically significant. ADR: adverse drug reaction; PTB: pulmonary tuberculosis; EPTB: extrapulmonary tuberculosis; DS: drug-sensitive; DR: drug-resistant

Variables		Level of medication adherence			Total	Chi-square (χ²)	P-value
		High	Medium	Low			
Gender	Male	80 (63.49%)	19 (15.07%)	27 (21.42%)	126	12.45	0.002*
	Female	70 (72.16%)	22 (22.68%)	5 (5.15%)	97		
Age (years)	18-27	48 (64.86%)	12 (16.21%)	14 (18.91%)	74	14.36	0.157
	28-37	49 (62.82%)	17 (21.79%)	12 (15.38%)	78		
	38-47	23 (76.66%)	4 (13.33%)	3 (0.10%)	30		
	48-57	17 (73.91%)	4 (17.39%)	2 (8.69%)	23		
	58-67	12 (92.30%)	1 (7.69%)	0	13		
	>68	1 (20%)	3 (60%)	1 (20%)	5		
Diagnosis	DS-TB	92 (67.64%)	23 (16.91%)	21 (15.44%)	136	0.7088	0.70158
	DR-TB	58 (66.66%)	18 (20.68%)	11 (12.64%)	87		
	PTB-DS	55 (71.42%)	10 (12.98%)	12 (15.58%)	77	1.41	0.493
	PTB-DR	45 (65.21%)	14 (20.28%)	10 (14.49%)	69		
	EPTB-DS	33 (61.11%)	13 (24.07%)	8 (14.81%)	54	2.63	0.268
	EPTB-DR	11 (78.57%)	3 (21.42%)	0	14		
	Disseminated TB-DS	5 (83.30%)	0	1 (16.70%)	6	3.0	0.223
	Disseminated TB-DR	1 (33.30%)	1 (33.30%)	1 (33.30%)	3		
ADR	Occurred	65 (65.65%)	17 (17.17%)	17 (17.17%)	99	1.1992	0.54902
	Not occurred	85 (68.54%)	24 (19.35%)	15 (12.09%)	124		

**Figure 4:**
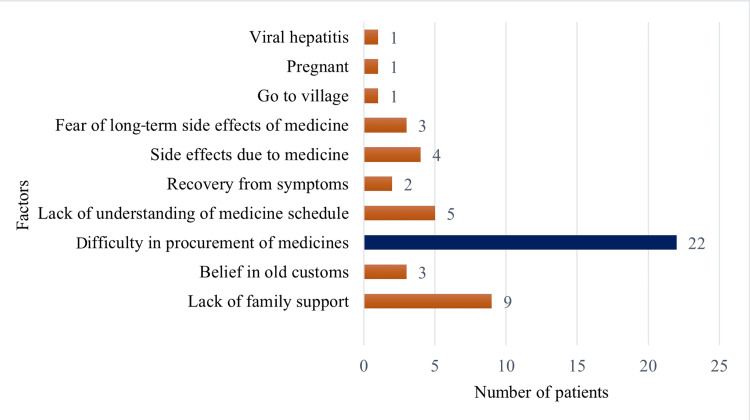
Factors affecting medication adherence

The most frequently reported factors affecting medication adherence included difficulty in procurement of medicines (22, 9.86%), lack of family support (nine, 4.03%), lack of understanding of the medicine schedule (five, 2.24%), and side effects (four, 1.8%). Other factors such as belief in traditional customs, recovery from symptoms, fear of long-term side effects, travel to villages, pregnancy, and co-infection with viral hepatitis were also mentioned (Figure 4).

**Figure 5:**
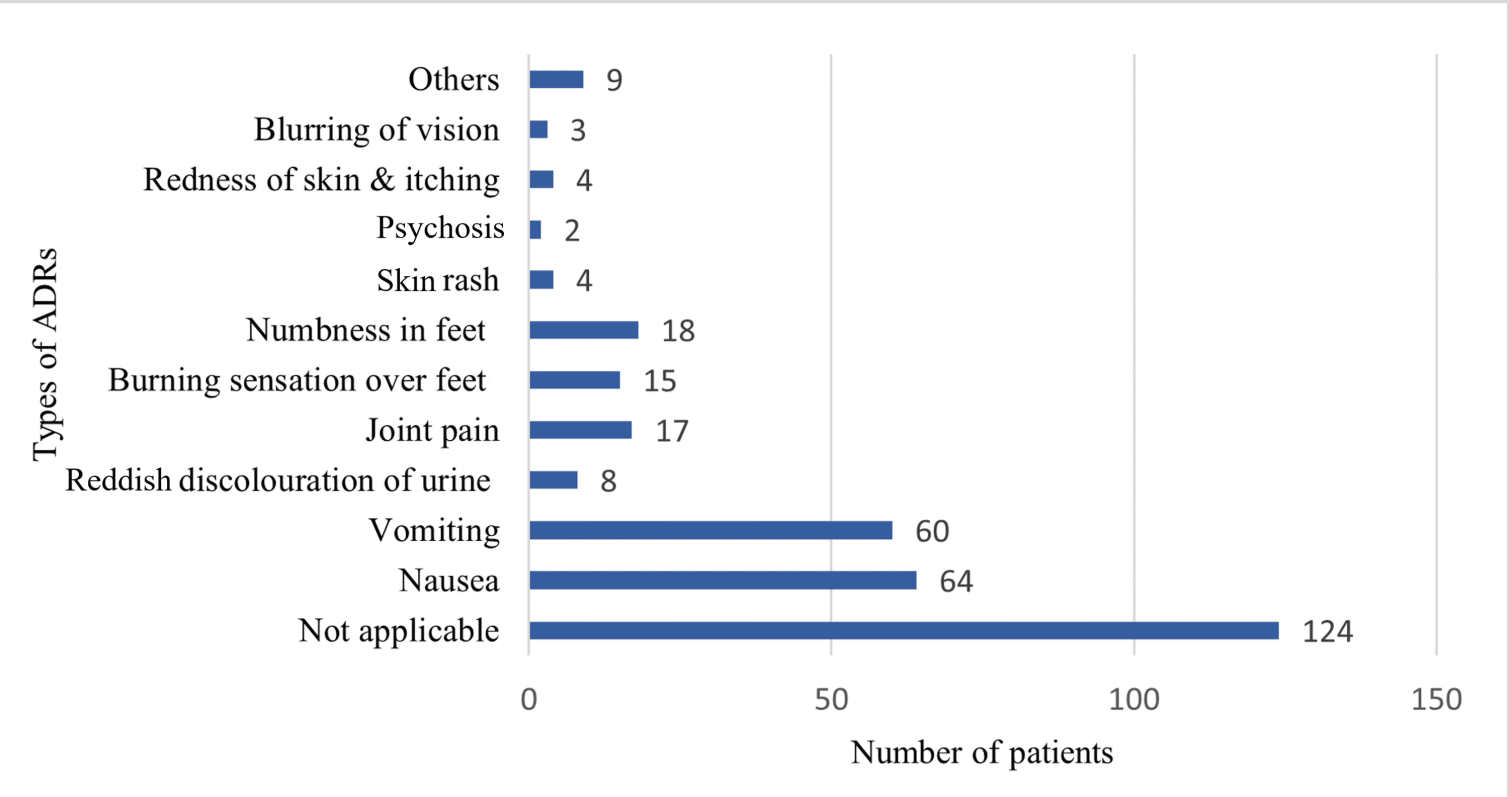
ADRs ADRs: adverse drug reactions

Adverse drug reactions (ADRs) of medication were reported by many patients. Among the total participants, 99 patients reported 128 ADRs during the study period, while the remaining 124 patients did not experience any side effects. The most frequently observed ADR was gastrointestinal (GI) upset, accounting for 60 (46.9%), making it the most prominent ADR. This was followed by peripheral neuropathy with 22 (17.2%) and arthralgia with 15 (11.7%). Other notable ADRs include reddish discolouration of urine and dermatological manifestations (e.g., rash, itching, redness), each reported in seven (5.5%) patients, whereas psychosis, headache, dizziness, and body ache were noted in two (1.6%) patients each. Less common ADRs (each with one reported case, or 0.78% of the total) included the following: optic neuritis, QT prolongation, prurigo simplex, Stevens-Johnson syndrome (SJS), generalized weakness, abdominal pain, breathlessness, menorrhagia, and blurring of vision (Figure 5).

## Discussion

The success of an anti-TB program largely depends on how effectively it is structured and implemented. In patients with chronic diseases, better medication adherence has been linked to better clinical outcomes, whereas worse medication adherence has been linked to more adverse events. Even though anti-TB drug regimens have significantly reduced morbidity and death, persistent infectiousness, a high risk of treatment failure, and the emergence of DR-TB could still happen if patients do not take their medications as prescribed. Many obstacles prevent patients from taking their medications; some of them can be overcome by the patient, while others may be institutional, structural, or otherwise beyond their control. Sadly, academics and healthcare professionals have a tendency to overlook operational and structural aspects of the environment in favor of viewing noncompliance as a patient issue. Therefore, tracking medication adherence and contributing factors is essential for program assessment and intervention [8].

In the present study evaluating treatment medication adherence among TB patients, several important associations between patient characteristics and medication adherence patterns were observed. Gender showed a statistically significant association with medication adherence levels (χ² = 12.45; p = 0.002), with female patients demonstrating higher medication adherence (70, 72.16% high medication adherence) compared to males (80, 63.49% high medication adherence). This aligns with the study of Munro et al., suggesting that women are generally more health-conscious and more likely to adhere to long-term treatment regimens [9]. These findings underscore the need for gender-sensitive strategies in TB programs, particularly interventions that address barriers faced by male patients, such as work-related migration, substance use, or stigma [10]. Although age did not show a statistically significant association with medication adherence (χ² = 14.36; p = 0.157), older patients (particularly those aged 58-67) demonstrated the highest proportion of high medication adherence (12, 92.3%), while younger patients (18-37 years) had lower medication adherence rates. This aligns with the study of Jing et al. indicating that older adults, who may perceive TB as a greater threat to health and have fewer competing social or work demands, often demonstrate better medication adherence [11]. These findings highlight the importance of age-specific interventions, including intensified counselling and support for younger patients who may face different practical and psychosocial barriers [12]. But the study of Bea et al. showed low medication adherence among elderly people [13].

Regarding clinical factors, medication adherence rates were remarkably similar across DS-TB and DR-TB groups with 92 cases (67.64%) vs. 58 cases (66.66%) reporting high medication adherence (χ² = 0.7088; p = 0.701). This contrasts with studies of Munro et al. and Kulkarni et al. that reported poorer medication adherence in DR-TB patients, attributed to longer, more toxic regimens and higher pill burden [9,10]. The comparable medication adherence in the present study likely reflects recent enhancements in programmatic support under the NTEP, including counselling, nutritional support, direct benefit transfers, and digital medication adherence technologies such as 99 Directly Observed Treatment Short-Course (DOTS) and Medication Event Reminder Monitor (MERM) boxes [14,15]. Additionally, no significant differences in medication adherence were found between PTB (χ² = 1.41; p = 0.493), EPTB (χ² = 2.63; p = 0.268), and disseminated TB (χ² = 3.00; p = 0.223) groups. The occurrence of ADRs did not significantly impact medication adherence (χ² = 1.1992; p = 0.549), despite ADR being a well-documented risk factor for non-adherence [16]. This may suggest the effective clinical management of ADRs, rapid regimen modifications when needed, and regular follow-up as recommended in NTEP guidelines [17].

In the present study, the most common patient-reported factors affecting medication adherence were difficulty in procurement of medicines, lack of family support, misunderstanding of the treatment schedule, and side effects. Additional factors included cultural beliefs, perceived recovery, fear of side effects, travel, pregnancy, and co-infection. In a study of Lolong et al., feeling better/there were no more symptoms, being declared cured by a healthcare professional, having no money, fear of side effects, and feeling no improvement are the common reasons for non-adherence [18]. In a study of Daksa et al., the common reasons for non-adherence are as follows: lack of family support, hospital is too far to follow up treatment regularly, due to side effects of medicine, and feeling better [8]. In a study of Karuniawati et al., the patient believed their medication was dangerous or harmful to them is the main reason for non-adherence [19].

GI symptoms were the most commonly reported ADRs in the present study which was similarly observed in different studies [20-22] as well. But in the study of Fei et al., cutaneous adverse drug reactions (CADRs) (44, 21%) were the most commonly reported ADRs [23]. The increased incidence of GI side effects could be attributed to multiple drug therapy as a major predisposing factor for ADRs.

Limitations of the study

As it was a cross-sectional study, no medication changes were noted, and only one-time data was obtained. Furthermore, medication adherence was assessed using the MMAS-8, which depends on patient self-reporting and may be subject to recall bias or social desirability bias.

## Conclusions

The present study among TB patients found overall good medication adherence, showing high medication adherence, particularly among females and older age groups. Medication adherence was similar between DS-TB and DR-TB cases, reflecting effective program support. The main barriers to medication adherence were difficulty in procurement of medicines, limited family support, and misunderstanding of treatment schedules. GI upset was the most frequently reported ADR, alongside neuropathy and arthralgia. These findings highlight the need for targeted patient education, reliable drug supply, and proactive management of side effects to further strengthen TB treatment medication adherence and outcomes.

## Figures and Tables

**Figure 6:**
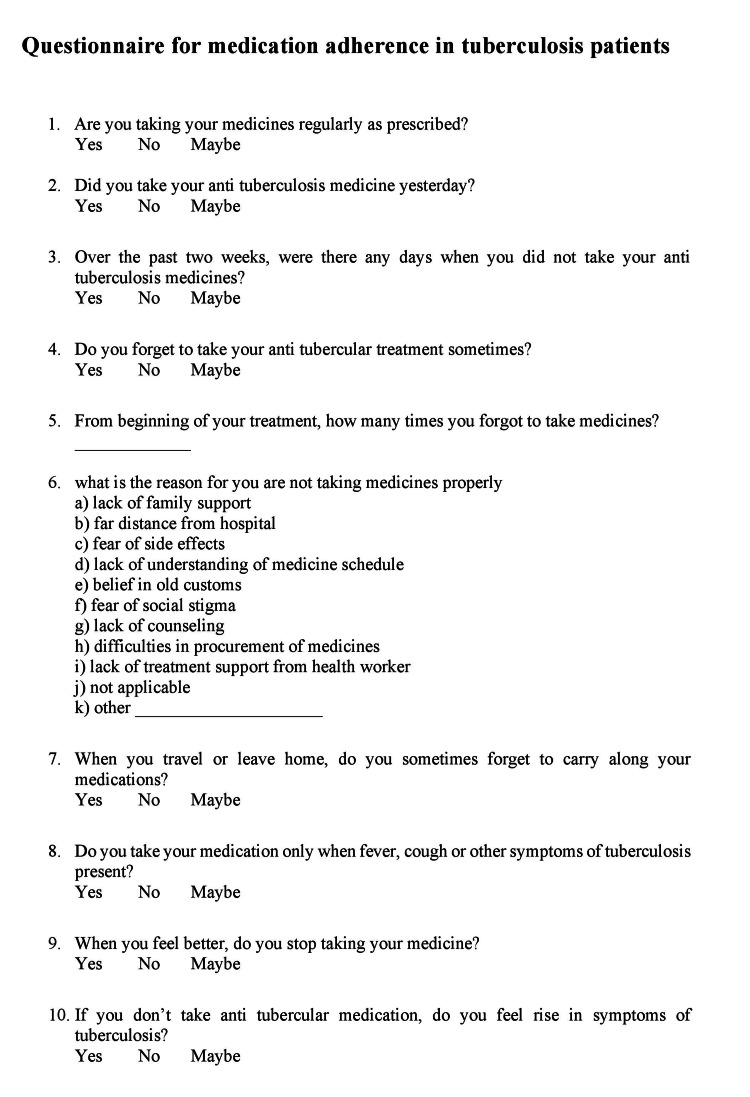
Questionnaire for medication adherence in tuberculosis patients

**Figure 7:**
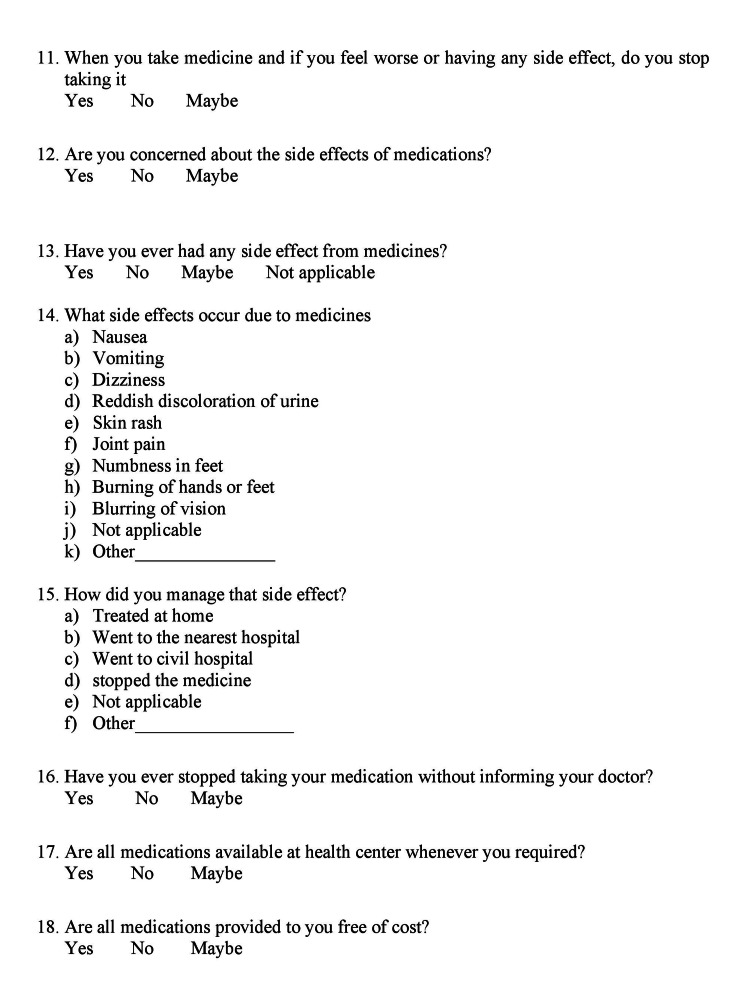
Questionnaire for medication adherence in tuberculosis patients (continuation)
